# 952. Implementation of Antimicrobial Stewardship Weekend Coverage within a Community Hospital with a PGY2 Infectious Diseases Pharmacy Resident

**DOI:** 10.1093/ofid/ofac492.795

**Published:** 2022-12-15

**Authors:** Lacy Worden, Lisa E Dumkow, C Ryan Tomlin, Nnaemeka Egwuatu

**Affiliations:** Mercy Health Saint Mary's, Lowell, Michigan; Trinity Health Saint Mary's Grand Rapids, Grand Rapids, MI; Mercy Health Saint Mary's, Lowell, Michigan; Mercy Health Saint Mary's, Lowell, Michigan

## Abstract

**Background:**

Antibiotic use contributes to antibiotic resistance affecting individual patients and communities. Important antimicrobial stewardship program (ASP) strategies, such as prospective audit and feedback, may be limited to peak weekday hours in many institutions. Currently, a lack of data exists to support expansion of ASP beyond peak hours in community hospitals. The goal of this study was to describe the impact of expanding inpatient ASP weekend coverage with a newly established PGY2 infectious diseases pharmacy resident program.

**Methods:**

This retrospective cohort study was approved by the Institutional Review Board and conducted using the pharmacist documentation function within the electronic health record of weekend interventions taking place between July 1, 2021 and December 31, 2022. The primary objective was to describe the impact of expanding weekend AMS coverage with a PGY2 ID pharmacy resident through quantification of inpatient antimicrobial stewardship interventions. Secondary endpoints included comparing the PGY2 resident’s weekend stewardship activities based on intervention quantity, type, and impact, to weekends without PGY2 ID resident coverage. Comparator groups included: 1) A single experienced clinical pharmacist, 2) Two new PGY1 pharmacy residents (first 6 months of residency), and 3) Two experienced PGY1 pharmacy residents (final 6 months of residency).

**Results:**

8 weekends of interventions were collected for each group. Significantly more interventions, including low-, medium-, and high-impact interventions were made by an ID-PGY2 pharmacy resident compared to a clinical pharmacist and both PGY1 groups. The median number of interventions made by each group are outlined in Table 1. Additionally, significantly more interventions were made per protocol as well as interventions that required communication with providers.

**Figure:**
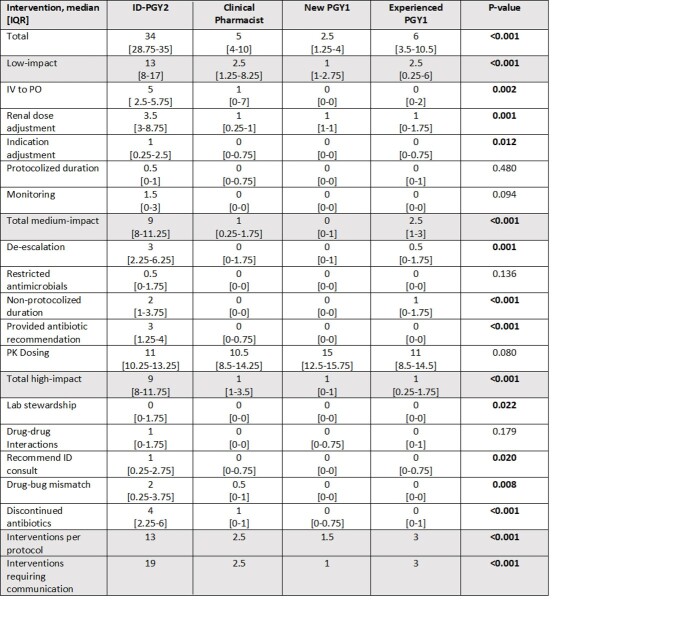

**Conclusion:**

Expansion of ASP services to include weekend clinical coverage with an ID PGY2 pharmacy resident significantly increased weekend ASP interventions in a community teaching hospital. Specialty pharmacy residency training programs may offer a high-value opportunity to expand ASP coverage.

**Disclosures:**

**All Authors**: No reported disclosures.

